# Effect of avian influenza A H5N1 infection on the expression of microRNA-141 in human respiratory epithelial cells

**DOI:** 10.1186/1471-2180-13-104

**Published:** 2013-05-10

**Authors:** Wai-Yip Lam, Apple Chung-Man Yeung, Karry Lei-Ka Ngai, Man-Shan Li, Ka-Fai To, Stephen Kwok-Wing Tsui, Paul Kay-Sheung Chan

**Affiliations:** 1 School of Biomedical Sciences, Faculty of Medicine, The Chinese University of Hong Kong, New Territories, Hong Kong Special Administration Region, Shatin, People’s Republic of China; 2 Departments of Microbiology, Faculty of Medicine, The Chinese University of Hong Kong, Prince of Wales Hospital, New Territories, Hong Kong Special Administration Region, Shatin, People’s Republic of China; 3 Department of Anatomical and Cellular Pathology, Faculty of Medicine, The Chinese University of Hong Kong, Prince of Wales Hospital, New Territories, Hong Kong Special Administration Region, Shatin, People’s Republic of China; 4 Stanley Ho Centre for Emerging Infectious Diseases, Faculty of Medicine, The Chinese University of Hong Kong, Prince of Wales Hospital, New Territories, Hong Kong Special Administration Region, Shatin, People’s Republic of China

**Keywords:** microRNA, Influenza A virus, H1N1, H5N1, Inflammation, Hypercytokinemia, Pathogenesis

## Abstract

**Background:**

Avian influenza remains a serious threat to human health. The consequence of human infection varies markedly among different subtypes of avian influenza viruses. In addition to viral factors, the difference in host cellular response is likely to play a critical role. This study aims at elucidating how avian influenza infection perturbs the host’s miRNA regulatory pathways that may lead to adverse pathological events, such as cytokine storm, using the miRNA microarray approach.

**Results:**

The results showed that dysregulation of miRNA expression was mainly observed in highly pathogenic avian influenza A H5N1 infection. We found that miR-21*, miR-100*, miR-141, miR-574-3p, miR-1274a and miR1274b were differentially expressed in response to influenza A virus infection. Interestingly, we demonstrated that miR-141, which was more highly induced by H5N1 than by H1N1 (*p* < 0.05), had an ability to suppress the expression of a cytokine - transforming growth factor (TGF)-β2. This was supported by the observation that the inhibitory effect could be reversed by antagomiR-141.

**Conclusions:**

Since TGF-β2 is an important cytokine that can act as both an immunosuppressive agent and a potent proinflammatory molecule through its ability to attract and regulate inflammatory molecules, and previous report showed that only seasonal influenza H1N1 (but not the other avian influenza subtypes) could induce a persistent expression of TGF-β2, we speculate that the modulation of TGF-β2 expression by different influenza subtypes via miR-141 might be a critical step for determining the outcome of either normal or excessive inflammation progression.

## Background

Avian influenza remains a serious threat to poultry and human health. From December 2003 to April 2013, more than 600 human infections and 374 deaths have been reported to the World Health Organization [1]. Outbreaks of H5N1 in poultry swept from Southeast Asia to many parts of the world. To date, there is still no sign that the epidemic is under control.

While it has been well documented that infection with H5N1 results in high mortality in humans [2-5], the cellular pathway leading to such adverse outcome is unknown. The naive host immune system cannot be the sole explanation as infection of other avian influenza viruses, e.g. H9N2, only results in mild infections [6]. While the predilection of H5N1 towards cells in the lower respiratory tract contributes to the development of severe pneumonia [7], the available clinico-pathological evidence indicates that the infected patients progress to multi-organ failure early in the course of illness, and the degree of organ failure is out of proportion to the involvement of infection [8-10]. Cytokine storm and reactive haemophagocytic syndrome are the key features that distinguish H5N1 infection from severe seasonal influenza. These indirect mechanisms seem to play an even more important role than direct cell killing due to lytic viral infection.

MiRNAs, a new class of endogenous, 18–23 nucleotide long noncoding and single-stranded RNAs, were recently discovered in both animals and plants. They trigger translational repression and/or mRNA degradation mostly through complementary binding to the 3′UTR of target mRNAs. Studies have shown that miRNAs can regulate a wide array of biological processes such as cell proliferation, differentiation, and apoptosis [11-14].

Given the nature of viruses, being intracellular parasites and using the cellular machinery for their survival and replication, the success of the virus essentially depends on its ability to effectively and efficiently use the host machinery to propagate itself. This dependence on the host also makes it susceptible to the host gene-regulatory mechanisms, i.e. the host miRNAs may also have direct or indirect regulatory role on viral mRNAs expression.

Recently, several reports indicated that miRNAs can target influenza viruses and regulate influenza virus replication. In one report, 36 pig-encoded miRNAs and 22 human-encoded miRNAs were found to have putative targets in swine influenza virus and Swine-Origin 2009 A/H1N1 influenza virus genes, respectively [15]. In another report, results showed that miR-323, miR-491 and miR-654 could inhibit replication of H1N1 influenza A virus through binding to the conserved region of the PB1 gene [16]. These miRNAs could downregulate PB1 expression through mRNA degradation instead of translation repression [16]. Besides targeting influenza virus, cellular miRNAs were also implicated in the lethal infections of mice with a highly pathogenic 1918 pandemic H1N1 influenza virus [17]. A previous study on miRNA gene expression in avian influenza virus infected chicken showed that miR-146, which was previously reported to be associated with immune-related signal pathways in mammals, was found to be differentially expressed in infected tissues [18]. Moreover, a study of profiling cellular miRNAs of lung tissue from cynomolgus macaques infected with a highly pathogenic H5N1 avian and a less pathogenic 1918 H1N1 reassortant virus identified that 23 miRNAs were associated with the extreme virulence of highly pathogenic H5N1 avian virus [19]. Also, the predicted gene targets of the identified miRNAs were found to be associated with aberrant and uncontrolled inflammatory responses and increased cell death [19].

This study aimed at elucidating how avian influenza infection perturbs the human gene regulatory pathways leading to adverse pathological events, e.g. cytokine storm. We hypothesized that miRNAs could be involved in influenza virus infection response and began addressing this hypothesis using a microarray-based screening. The ultimate goal of this study is to generate essential information for further studies to identify novel intervention targets to ameliorate the adverse outcome of infection.

## Results

### Differential miRNA expression in H5N1 and H1N1 influenza virus infected cells

The cell line - NCI-H292, infected with various preparations of influenza viruses was analysed for miRNA expression profiles subsequently. A list of differentially expressed miRNA was identified for subtypes H1N1 and H5N1, respectively (Table 1), and the temporal pattern of expression was delineated. Among the listed profiles of differentially up-regulated miRNA, it was found that miR-141, miR-181c*, miR-210, miR29b, miR-324-5p, and miR-663 were up-regulated (>1.5-fold, p<0.05) at 3-hour post-infection with subtype H5 as compared with non-infected control cells. At this time point, only miR-141 was found to be slightly induced in subtype H1 infected cells. At 6-hour post-infection, it was found that miR-483-3p was up-regulated (>3-fold, p<0.05) in H5N1 infected cells while miR-663 was found to be up-regulated (>1.5-fold, p<0.05) in H1N1 infected cells. At 18 and 24-hour post-infection, miR-923, miR-1246, miR-574-3p, and miR-663 were up-regulated (>3-fold, p<0.05) in H5N1 infected cells. For H1N1 infected cells, at 18 and 24-hour post-infection, miR-188-5p, miR-1260, miR-1274a, miR-1274b, miR141, miR183*, miR-18b, miR-19a, miR21*, miR-301a, miR-572, miR-720, and miR-939 were found to be up-regulated (>1.5-fold, p<0.05) (Table 1).

**Table 1:** miRNAs differentially expressed in H1N1 and H5N1 infected NCI-H292 cells at different time points, respectively

**A) MiRNAs differentially expressed in cells infected with H1N1 influenza A virus at 3, 6, 18, and 24 hours post-infection, respectively**											
**3-hour**	**Fold-change**	**Regulation**	**6-hour**	**Fold-change**	**Regulation**	**18-hour**	**Fold-change**	**Regulation**	**24-hour**	**Fold-change**	**Regulation**
hsa-miR-141	1.51	up	hsa-miR-663	1.59	up	hsa-miR-188-5p	1.57	up	hsa-miR-1260	1.58	up
hsa-miR-23a	2	down	hsa-miR-15a*	1.61	down	hsa-miR-1260	1.77	down	hsa-miR-1274a	1.66	up
hsa-miR-574-3p	2.83	down	hsa-miR-1825	1.51	down	hsa-miR-1274a	1.86	down	hsa-miR-1274b	1.91	up
hsa-miR-574-5p	2.99	down	hsa-miR-183*	1.71	down	hsa-miR-1274b	1.69	down	hsa-miR-141	1.51	up
			hsa-miR-34b	1.52	down	hsa-miR-141	1.66	down	hsa-miR-183*	1.54	up
			hsa-miR-494	1.56	down	hsa-miR-17*	1.927	down	hsa-miR-18b	1.64	up
			hsa-miR-574-5p	1.74	down	hsa-miR-21*	1.71	down	hsa-miR-19a	1.52	up
									hsa-miR-21*	1.7	up
									hsa-miR-301a	1.53	up
									hsa-miR-572	1.5	up
									hsa-miR-720	1.99	up
									hsa-miR-939	1.51	up
									hsa-miR-181c*	1.53	down
**B) MiRNAs differentially expressed in cells infected with H5N1 influenza A virus at 3, 6, 18, and 24 hours post-infection, respectively.**											
has-miR-141	1.9	up	hsa-miR-483-3p	3.06	up	hsa-miR-188-5p	2.01	up	hsa-miR-1181	2.6	up
hsa-miR-181c*	1.8	up	hsa-miR-let-7b*	2.02	up	hsa-miR-923	3.39	up	hsa-miR-1207-5p	2.7	up
hsa-miR-210	1.5	up	hsa-miR-126	2.2	down	hsa-miR-1260	3.11	down	hsa-miR-1224-5p	2.02	up
hsa-miR-29b	1.62	up	hsa-miR-20a*	2.42	down	hsa-miR-1274a	3.57	down	hsa-miR-1225-5p	2.44	up
hsa-miR-324-5p	1.759	up	hsa-miR-362-5p	2.6	down	hsa-miR-1274b	4.61	down	hsa-miR-1246	4.39	up
hsa-miR-663	2.01	up	hsa-miR-378	2.16	down	hsa-miR-141	3.2	down	hsa-miR-134	2.78	up
hsa-miR-197	1.64	down	hsa-miR-454	2.32	down	hsa-miR-18a	2.15	down	hsa-miR-188-5p	2.49	up
hsa-miR-339-3p	1.925	down	hsa-miR-574-5p	2.02	down	hsa-miR-18b	3.34	down	hsa-miR-1915	2.84	up
hsa-miR-574-3p	1.77	down				hsa-miR-19a	2.32	down	hsa-miR-572	2.92	up
hsa-miR-574-5p	2.41	down				hsa-miR-21*	3.23	down	hsa-miR-574-3p	3.75	up
						hsa-miR-301a	2.32	down	hsa-miR-574-5p	2.083	up
						hsa-miR-30e	2.24	down	hsa-miR-629*	2.85	up
						hsa-miR-720	3.39	down	hsa-miR-638	2.19	up
									hsa-miR-663	4.52	up
									hsa-miR-939	2.32	up
									hsa-miR-100*	3.47	down
									hsa-miR-1260	3.09	down
									hsa-miR-1280	3.01	down
									hsa-miR-141	4.5	down
									hsa-miR-21*	4	down
									hsa-miR-221	2.72	down
									hsa-miR-455-3p	2.16	down

Among the listed profiles of differentially down-regulated miRNA as compared with non-infected control cells, it was found that miR-574-5p was down regulated (>2-fold, p<0.05) in H5N1 infected cells at 3-hour post-infection. For H1N1 infected cells, miR-23a, miR-574-3p and miR-574-5p were down-regulated (>=2-fold, p<0.05) at this time point. At 6-hour post-infection, miR-126, miR-20a*, miR-362-5p, miR-378, miR-454, and miR574-5p were found to be down-regulated (>2-fold, p<0.05) in H5N1 infected cells. At the same time point (6-hour), miR-15a*, miR-1825, miR-183*, miR-34b, miR-494, and miR-574-5p were found to be down-regulated (>1.5-fold, p<0.05) in H1N1 infected cells. Furthermore, at 18, and 24-hour post-infection, miR-1260, miR-1274a, miR-1274b, miR-141, miR-18b, miR-21*, miR-720, miR-100*, miR-1260, miR1280, and miR21* were found to be down-regulated (>3-fold, p<0.05) in H5N1 infected cells. At these time points, only miR-1274, and miR-17* were found to be down-regulated (>1.8-fold, p<0.05) in H1N1 infected cells (Table 1).

From the results, we found that similar changes in miRNA profiles were observed in both H1N1 and H5N1 infection. However, the magnitude of fold-changes which occurred in H1N1 infection were much lower than that in H5N1 infection.

### Confirmation of miRNA expression profile by real-time PCR

**Figure 1:**
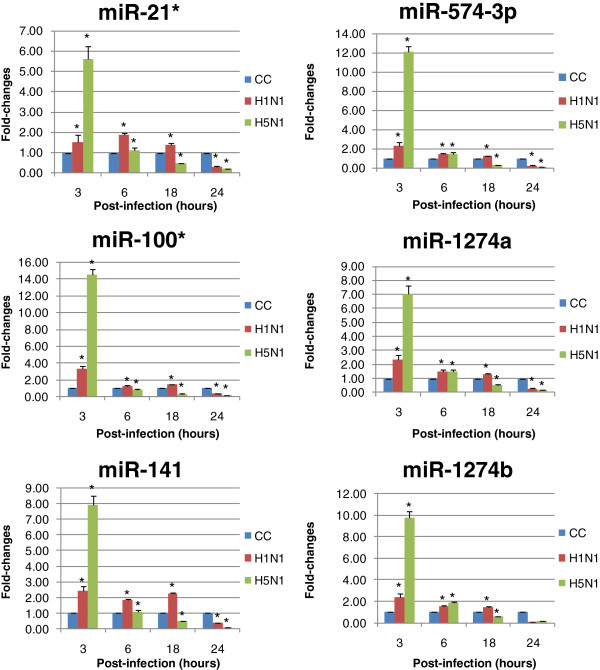
**Patterns of changes in cellular miRNA expression after influenza A virus infection.** NCI-H292 cells were infected with influenza A virus subtypes: H1N1/2002 or H5N1/2004 viruses at m.o.i. = 1, respectively. qRT-PCR were used to quantitify the miRNA levels and fold-changes were calculated by ΔΔCT method as compared with non-infection cell control (CC) and using 18S rRNA level for normalization. Each point on the graph represents the mean of fold-changes. The mean fold-changes of miRNA in H1N1 or H5N1 infected cells were compared to that of non-infected controls ± SD (p* < 0.05).

The microarray data were further confirmed using TaqMan quantitative RT-PCR (qRT-PCR) assays. There were general consistency between TaqMan qRT-PCR assays and microarray results. It was found that six miRNAs (miR-21*, miR-100*, miR-141, miR-1274a, miR-1274b and miR-574-3p) were initially up-regulated at 3 hours post-infection. The degree of up-regulation was more prominent in H5N1 infection (5 to 14 folds)(p*<0.05) than in H1N1 infection (1.5 to 3 folds)(p*<0.05). It was also found that these miRNAs became down-regulated during 6-to-24 hours post-infection. The degree of down-regulation was also higher in H5N1 infection than in H1N1 infection (Figure 1).

### Target prediction of the miRNA expression profile

We then examined the list of targets predicted by TargetScan computer software ( http://www.targetscan.org/) for the miRNA species that had the most consistent and significant changes in expression following influenza A virus infection (Table 2) [20]. The TargetScan results showed that many of the target genes were involved in the inflammatory response and cell death pathways. Interestingly, one of the target prediction results showed that there was a 3′ untranslated region (UTR) binding site on TGF-β2 for miR-141. The miR-141 sequence is: 3′- GGUAGAAAUGGUCU***GUCACAAU*** - 5′, while that of TGF-β2 3′UTR is: 5′-AGAGCCUUGGUUCAU***CAGUGUUA***-3′. We had previously reported that TGF-β2 was an important cytokine involved in the inflammatory response of avian influenza A virus infection [21] and, together with the results showing that the expression of miR-141 was altered during the time course of influenza A virus infection, we selected miR-141 for further functional analysis in this study.

**Table 2:** The potential targets of selected miRNA: miR-21*, miR-100*, miR-141, miR-1274a, miR-1274b, and miR-574 -3p are listed

**miRNA**	**Gene name**	**Predicted target site**
miR-21*	CCL17	Small inducible cytokine A17 precursor
	IL22	Interleukin-22 precursor
	C2orf28	Apoptosis-related protein 3 precursor
	TNFSF13	Tumor necrosis factor ligand superfamily member 12
	CCL1	Small inducible cytokine A1 precursor
	CCL19	Small inducible cytokine A19 precursor
miR-100*	IL13RA1	Interleukin-13 receptor alpha-1 chain precursor (IL-13R-alpha-1)
	CYTL1	Cytokine-like protein 1 precursor
	IL18RAP	Interleukin-18 receptor accessory protein precursor
**miR-141**	CXCL12	chemokine (C-X-C motif) ligand 12 (stromal cell-derived factor 1)
	TGFB2	transforming growth factor, beta 2
	CRLF3	cytokine receptor-like factor 3
	IFNAR1	interferon (alpha, beta and omega) receptor 1
**miR-574-3p**	NDUFA4L2	NADH dehydrogenase (ubiquinone) 1 alpha subcomplex, 4-like 2
**miR-1274a**	TNFAIP3	tumor necrosis factor, alpha-induced protein 3
	TNFAIP8L2	tumor necrosis factor, alpha-induced protein 8-like 2
	BCL2L2	BCL2-like 2
	BCLAF1	BCL2-associated transcription factor 1
	BCLAF1	BCL2-associated transcription factor 1
**miR-1274b**	TNFAIP8L2	tumor necrosis factor, alpha-induced protein 8-like 2
	IL1RAPL1	interleukin 1 receptor accessory protein-like 1
	BCLAF1	BCL2-associated transcription factor 1

### MiR-141 represses the expression of TGF-β2 mRNA

**Figure 2:**
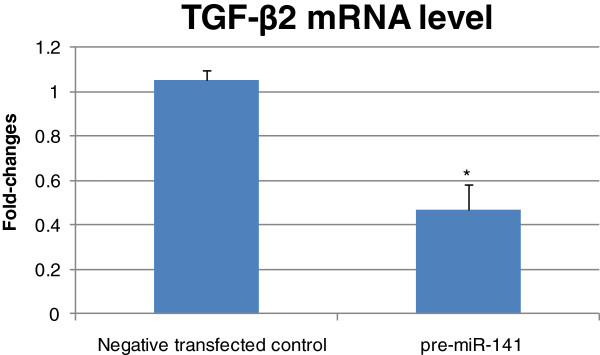
**The TGF-β2 3′UTR is regulated by miR-141.** NCI-H292 cells were transfected with pre-miR-141 and negative control, respectively. The fold-changes of mRNA level of TGF-β2 as measured by qRT-PCR at 24 hours after transfection. Fold-changes were calculated by ΔΔCT method as compared with negatively transfected cell control and using β-actin level for normalization. Each point on the graph represents the mean fold-changes. The mean fold-changes of TGF-β2 mRNA level was compared to that of negative control ± SD (p* < 0.05).

In addition to the miRNA target prediction results, by using ecoptic expression of miR-141, the level of TGF-β2 mRNA was found to be significantly decreased in miR-141 transfected cells but not in negative-control miRNA mimic transfected cells (Figure 2). In this over-expression system we could determine that the 3′UTR was the miR-141 target and the decreased TGF-β2 mRNA level might be due to the binding of miR-141 to the 3′UTR of TGF-β2 mRNA which reduced the half-lives of TGF-β2 mRNA.

### Effect of inhibition of miR-141 in influenza A virus infection

**Figure 3:**
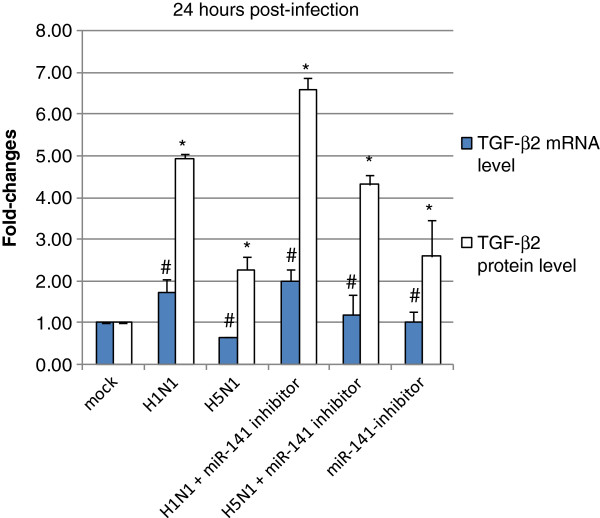
**Measurement of TGF-β2 mRNA and protein level.** NCI-H292 cells with or without treatment of miR-141 inhibitor, were infected with influenza A virus subtypes: H1N1/2002 or H5N1/2004 viruses at m.o.i. = 1, respectively for 24 hours. qRT-PCR were used to quantitify the TGF-β2 mRNA levels and fold-changes were calculated by ΔΔCT method as compared with non-infection cell control (mock) and using endogeneous actin mRNA level for normalization. TGF-β2 protein level was measured by enzyme-linked immunosorbent assay as compared with mock. Each point on the graph respresents the mean fold-changes. The experimental mean fold-changes of mRNA and protein levels were compared to that of mock controls ± SD (p* < 0.05), (p^#^< 0.05), respectively.

The functional relevance of changes in miR-141 expression during influenza A virus infection was assessed using miRNA inhibitors. Chemically modified, single stranded nucleic acids anti-miR miR-141 inhibitor and negative control were transfected into H292 cells for 24 hours. We had previously shown that this was sufficient time to obtain oligonucleotide delivery in H292 cells when examining the inhibition of TGF-β2 mRNA expression. After the cells were pre-treated with anti-miR miR-141 for 24 hours, they were then infected with H1N1 or H5N1, respectively. After the infection processes, anti-miR miR-141 was transfected again into the virus infected cells and incubated for another 24 hours. The results of this experiment showed that the anti-miR miR-141 inhibitor could cause an increase in TGF-β2 protein expression in H1N1 or H5N1 infected cells, as compared to cells only infected with H1N1 or H5N1 but without anti-miR miR-141 inhibitor treatment (Figure 3). The effect was also more prominent in H5N1 infection than that of H1N1.

## Discussion

In this study we examined the connection between influenza A virus infection and the global patterns of cellular miRNA expression. The major observations from this work were that influenza A virus infection resulted in the altered regulation of cellular miRNAs. Avian influenza A virus can alter cellular miRNAs to a greater extent than that of seasonal human influenza A virus.

Influenza A virus affects the regulation of many cellular processes. In some cases, these changes are directed by the virus for its advantage and others are cellular defense responses to infection. Here, we found that influenza A virus infection led to altered regulation of cellular miRNAs. Given the number of genes that can be regulated by individual miRNAs and the number of miRNAs expressed in cells, this greatly expands the range of possible virus-host regulatory interactions. The complexity is underscored by there being no uniform global pattern of regulation; rather, it appears that individual (or groups of) miRNA are independently regulated, some positively and some negatively. Persistent and transient effects were seen, and changes in miRNA expression profiles were linked to the time course of infection. As a summary, miR-1246, miR-663 and miR-574-3p were up-regulated (>3-fold, p<0.05) at 24-hour post-infection with subtype H5 as compared with non-infected control cells. Moreover, miR-100*, miR-21*, miR-141, miR-1274a and miR1274b were found to be down-regulated (>3-fold, p<0.05) in infection with subtype H5, particularly at 18 or 24 hours post-infection as compared with non-infected control cells. Interestingly, many of the virally regulated miRNAs were predicted by TargetScan to target important biological pathways, immune-related signal pathways and have altered regulation in some cellular defense and some states of cellular differentiation [22].

In our study, we found that the expression of miR-141 was affected by influenza A virus infection. To validate the in silico findings empirically on the target of miR-141, we checked whether transient-transfection of anti- and pre-mir-141 into NCI-H292 cells resulted in TGF-β2 regulation. In our experiment, the transfection efficiency was an important factor affecting the degree of regulation on the target gene(s). In the case of higher transfection efficiency, as more miRNA would be transfected into the cells, the effect of gene(s) regulation by miRNA transfected would be greater. In our study, the transfection efficiency was about 78.2 ± 6.3% (mean ± SD), which was considered to be adequate for further functional analyses. During transfection, some oligonucleotide molecules were sequestered in internal vesicles and physically separated from their targets in the cytoplasm; and then released during cell lysis. Therefore monitoring miRNAs by qPCR after transfection would not be valuable. Previous researchers of this procedure had highly recommended investigating the target mRNAs and proteins instead of miRNA quantification. The time point of 24-hour post-transfection or post-infection was chosen for evaluation because miR-141 induction was observed at the early stage of virus infection, and sufficient time might be required for the miR-141 to have effect on its target(s), so we had chosen 24-hour post-transfection or post-infection for evaluation of the effect of this miRNA.

Indeed, upon detecting the TGF-β2 expression at mRNA and protein levels, we found that the altered miR-141 expression would affect the expression of the cytokine- TGF-β2. Literature search on the background of miR-141 confirmed that miR-141 is a member of the miR-200 family (miR-200a, miR-200b, miR-200c, miR-141 and miR-429). Previous studies of miR-141 were mainly on its role in cancer. It has been reported that miR-141 were markedly downregulated in cells that had undergone epithelial to mesenchymal in response to TGF-β. MiR-141 was also found to be overexpressed in ovarian and colorectal cancers [23,24] and down-regulated in prostate, hepatocellular, renal cell carcinoma and in gastric cancer tissues [25-28] raising a controversial issue about the role of miR-141 in cancer progression. Furthermore, the miR-200 family members play roles in maintaining the epithelial phenotype of cancer cells [29]. A member of this family - miR-200a was also found to be differentially expressed in response to influenza virus infection in another study [17]. The targets of miR-200a are associated with viral gene replication and the JAK-STAT signaling pathway, which is closely related to type I interferon-mediated innate immune response [17]. However, the effect of miR-141 on virus infection was not known, except one recent report showing that enterovirus can induce miR-141 and contribute to the shutoff of host protein translation by targeting the translation initiation factor eIF4E [30].

In addition, evidence suggests that influenza A virus infection reduces or promotes the expression of the host miR-141 in a time dependent manner. We found that TGF-β2 mRNA was suppressed in miR-141 overexpressed cells. Our observation is in line with another study showing that the 3′UTR of TGF-β2 mRNA contained a target site for miR-141/200a and the expression of TGF-β2 was significantly decreased in miR-141/200a transfected cells [22]. Furthermore, miR-141 may not only work as translational repressors of target mRNAs, because it was observed that they also caused a decrease in TGF-β2 mRNA levels. These findings are similar to recent data demonstrating that some miRNAs can alter the mRNA levels of target genes [31]. This ability is probably independent of the ability of these miRNAs to regulate the translation of target mRNAs [14].

We also noted that antagomiR-141 moderately increased the accumulation of TGF-β2 protein during influenza virus infection. This might be because, by the use of anti-miR miR-141 inhibitor, which decreases the cellular pool of miR-141, the translation control of the TGF-β2 mRNA was subsequently released and caused the TGF-β2 protein to express and accumulate during virus infection. However, it was also observed that when there was an increase in TGF-β2 mRNA level, the corresponding TGF-β2 protein expression level would be increased, except in the case of non-miR-141-inhibitor treated H5N1 infected cells. In this case, there was a decrease in TGF-β2 mRNA level, while the TGF-β2 protein was increased. This might be explained by the fact that TGF-β2 mRNA degradation induced by miR-141 might be much faster than that of the corresponding protein degradation.

Recently, we had also reported that H1N1 was the only subtype that could induce a sustained increase in TGF-β2 at protein level [21]. That observation coincides with our results in this study, showing that H1N1 infection induced a little amount of miR-141 expression, while H5N1 infection induced a higher amount of miR-141 expression at the early phase of infection. As a consequence of the higher amount of miR-141 in H5N1 infection, TGF-β2 expression might be more greatly reduced than that in H1N1 infection. Since TGF-β2 can act as both an immunosuppressive agent and a potent proinflammatory molecule through its ability to attract and regulate inflammatory molecules, it plays a vital role in T-cell inhibition. Furthermore, it has been reported that TGF-β2 inhibits Th1 cytokine-mediated induction of CCL-2/MCP-1, CCL-3/MIP-1α, CCL-4/MIP-1β, CCL-5/RANTES, CCL-9/MIP-1γ, CXCL-2/MIP-2, and CXCL-10/IP-10 [32]. Moreover, the pro-inflammatory responses during influenza A virus infection are tightly controlled by anti-inflammatory mediators, such as TGF-β2, to protect the easily damageable lung tissue from destructive side effects associated with virus induced inflammation. Therefore, the downregulation of TGF-β2 protein by miR-141 may be an important step in the excessive inflammation progression during influenza A virus infection, particularly in H5N1 infection. However, whether the recovery of TGF-β2 expression by anti-miR miR-141 inhibitor could resolve the hypercytokinemia stage of H5N1 infection needs to be further studied.

Although our findings were obtained from an *in vitro* model, we could apply these to the real situation of an *in vivo* model or tissue comprised of different cell types. In real bronchial environments, lung epithelial cells are the key target of influenza viruses [33,34]. After these cells are infected, they will activate an inflammatory cascade which launches a quick antimicrobial reaction and directs adaptive immunity to mount a protective response. Bronchial epithelial cells therefore modulate the activation of monocytes, macrophages, dendritic cells (DC), and T lymphocytes through cytokines and chemokines. Cytokines and chemokines generally function in an autocrine (on the producing cell itself) or paracrine (on nearby cells) manner. These mediators will contribute to the generation of a specific bronchial homeostatic microenvironment that affects the way in which the body copes with the viruses. This homeostatic “circuit” can inhibit excessive inflammatory response in lung tissues [35]. For example, TGF-β had been reported to mediate a cross-talk between alveolar macrophages and epithelial cells [36]. However, our findings show that, during highly pathogenic H5N1 avian virus infection, miR-141 would be induced shortly after infection. With high level of miR-141, the expression of TGF-β would be suppressed from the lung epithelial cells. Without sufficient TGF- β, the pro-inflammatory response might not be tightly controlled in cases of highly pathogenic H5N1 avian virus infection. This might explain the mechanism concerning bronchial infiltration of inflammatory cells, particularly lymphocytes and eosinophils, and the subsequent hyperresponsiveness of the bronchial wall induced by viral infection.

Our study has some limitations that will need to be addressed in future studies. Firstly, we did not assess the roles of other miRNAs whose expression were also altered after infection. The miRNA microarrays that we used did not contain probes for every known miRNA; thus it is possible that influenza A virus infection affects the expression of some other miRNAs not yet covered by the kit used in the current study. Secondly, the virus may interact with miRNA regulatory pathways differently in other cell or tissue types, or in other physiological status.

## Conclusions

In conclusion, based on the broad-catching miRNA microarray approach, we found that dysregulation of miRNA expression is mainly observed in highly pathogenic avian influenza infection. We identified that miR-141 was induced at early time points upon influenza A virus infection. The induction was higher in H5N1 infection than that of seasonal H1N1 infection. Moreover, TGF-β2, which plays an important role in regulating inflammatory processes, was identified as a target of miR-141 binding. As a result, influenza A virus infection, in particular highly pathogenic H5N1, could affect the inflammatory processes via miR-141 induction.

## Methods

### Virus isolates

The influenza A H5N1 virus (A/Thai/KAN1/2004) (H5N1/2004) was isolated from a patient with fatal infection in Thailand in 2004. To serve as a comparison, a human seasonal H1N1 strain isolated in 2002 – (A/HongKong/CUHK-13003/2002) (H1N1/2002) was included. The research use of these samples was approved by the Joint CUHK - NTEC Research Ethics Committee, Hong Kong and the strains were isolated from the patients as part of standard care.

### Cell cultures

The bronchial epithelial cells - NCI-H292, derived from human lung mucoepidermoid carcinoma cells (ATCC, CRL-1848, Rockville, MD, USA), were grown as monolayers in RPMI-1640 medium (Invitrogen, Carlsbad, CA) supplemented with 10% fetal bovine serum (FBS), 100 U/ml penicillin and 100 μg/mL streptomycin (all from Gibco, Life Technology, Rockville, Md., USA) at 37°C in a 5% CO_2_ incubator. NCI-H292 cells were used as an *in- vitro* model to study host cellular responses to viral infection.

Mandin-Darby canine kidney (MDCK) cells were used for growing stocks of influenza virus isolates. MDCK cells were grown and maintained in Eagles Minimal Essential Media (MEM) containing 2% FBS, 100 U/ml penicillin and 100 μg/mL streptomycin (all from Gibco, Life Technology).

### Infection of cell culture with influenza A viruses

NCI-H292 cells were grown to confluence in sterile T75 tissue culture flasks for the inoculation of virus isolate at a multiplicity of infection (m.o.i.) of one. After 1 hour of absorption, the virus was removed and 2 ml of fresh RPMI-1640 media with 2% FBS, 100 U/ml penicillin, 100 μg/mL streptomycin and 1μg/ml L-1-tosylamido-2-phenylethyl chloromethyl ketone (TPCK)-treated trypsin (all from Gibco, Life Technology) was added, and incubated at 37°C in 5% CO_2_ humidified air.

### RNA extraction

Total RNA was extracted from normal and infected NCI-H292 cells using Trizol reagent (Invitrogen) following the manufacturer’s protocol. RNA pellets were resuspended in RNase-free water. The RNA integrity was assessed using Agilent 2100 Bioanalyzer (Agilent Technologies, Palo Alto, CA, USA).

### MiRNA expression profiling

MiRNAs were labeled using the Agilent miRNA labeling reagent and hybridized to Agilent human miRNA arrays according to the manufacturer’s protocol. Briefly, total RNA (100 ng) was dephosphorylated and ligated with 3′, 5′-cytidine bisphosphate (pCp-Cy3). Labeled RNA was purified and hybridized to Agilent miRNA arrays with eight identical arrays per slide, with each array containing probes interrogating 866 human miRNAs. Images were scanned with the Agilent microarray scanner (Agilent Technologies), gridded, and analyzed using Agilent feature extraction software (Agilent Technologies).

### Statistical analysis of microarray data

The cells were infected with either (A) the H1N1/2002 strain or (B) the H5N1/2004 strain, or (C) mock-infected with PBS (no infection control). Cell samples were collected at 3, 6, 18 and 24 hours post-infection. Each miRNA array allowed us to interrogate 866 human miRNAs. The results were analyzed using Genespring GX 10.0.2 software (Agilent Technologies). Firstly, the 16 arrays were quantile normalized together. Then, student’s paired *t*-test was applied to test if there was a significant difference between (A) the H1N1/2002-infected and (C) mock-infected, no infection control (matched for the time post-infection), (B) the H5N1/2004-infected and (C) mock-infected control, respectively. The resultant P-values were adjusted for multiple testing by using the Benjamini-Hochberg correction of the false-discovery rate [37]. MiRNAs with this adjusted P-value <= 0.05 were considered as differentially expressed. Those miRNAs, that are more than or equal to 3.5-fold up or down regulated were subjected to a second analysis using real-time RT-PCR.

### MicroRNA profiling data resource

The data discussed in this publication have been deposited in NCBI’s Gene Expression Omnibus [38] and are accessible through GEO Series accession number GSE44455.

### TaqMan Real Time RT-PCR (qRT-PCR) for quantification of miRNAs

Total RNA was reverse transcribed with looped miRNA-specific RT primers contained in the TaqMan MicroRNA assays ((Applied Biosystems, Foster City, CA). Briefly, single-stranded cDNA was synthesized from 10 ng total RNA in 15-μL reaction volume with TaqMan MicroRNA reverse transcription kit (Applied Biosystems), according to the manufacturer’s protocol. The reaction was incubated at 16°C for 30 min followed by 30 min at 42°C and inactivation at 85°C for 5 min. Each cDNA was amplified with sequence-specific TaqMan microRNA assays (Applied Biosystems). PCR reactions were performed on an Applied Biosystems Step One sequence detection system in 10 μl volumes at 95°C for 10 min, followed by 40 cycles of 95°C for 15 sec and 60°C for 1 min. All samples were tested in triplicate. The threshold cycle (Ct) values obtained with the SDS software (Applied Biosystems) were compared with the Ct obtained from 18S rRNA assay (Applied Biosystems) for the normalization of total RNA input. The fold-change was calculated based on Ct changes of mean medium Ct minus individual Ct of a miRNA. Each experiment was performed in triplicate.

### qRT-PCR for quantification of TGF-β2 mRNA level

Total RNA extracted from cell cultures was reversely transcripted to cDNA using the poly(dT) primers and Superscript III reverse transcriptase (Invitrogen), and quantified by real-time PCR. The sense and antisense primers used in real-time PCR for measuring TGF-β2 were: (Forward: 5′-CCAAAGGGTACAATGCCAAC-3′; Reverse: 5′-TAAGCTCAGGACCCTGCTGT-3′). The real-time PCR reactions were performed in triplicates using the SYBER Green PCR Master Mix (Applied Biosystems). The PCR conditions were 95 °C for 5 min, followed by 50 cycles of 95 °C for 30 sec, 55 °C for 30 sec, and 72 °C for 30 sec. The expression of β-actin gene was also quantified in a similar way for normalization. Comparative delta-delta C_T_ method was used to analyze the results where expression level of the respective gene at the corresponding time point in non-transfected cells was regarded as one [39,40]. Each experiment was performed in triplicate.

### Enzyme-linked immunosorbent assay (ELISA) measurement of TGF-β2 protein level

Cell culture supernatant was collected at 24 hours post-infection for the analysis of TGF-β2 expression. The total TGF-β2 protein level was measured by enzyme-linked immunosorbent assay (Emax® ImmunoAssay System, Promega, Madison, WI, USA) according to the manufacturer’s procedures. Each experiment was performed in triplicate.

### Reverse transfection of a mimic or an inhibitor of miR-141

The cells were transfected in suspension after trypsinisation with 60 nM anti-miR, pre-miR or negative control (Applied Biosystems). For the assay, 1x10^5^ cells per mL were transfected per well of a 24-well plate. Transfection complexes were prepared in OptiMEM (Invitrogen) with 1.5 μL/24-well of siPORT NeoFx transfection agent (Ambion, Austin, TX, USA). At 24 hours post-transfection, the cells were lysed for qRT-PCR analysis or subjected to H1N1 or H5N1 virus infection. The transfection efficiency was calculated from the percentage of fluorescent cells that were observed using florescence microscopy after the transfection of fluorescein isothiocyanate (FITC)-labeled short nucleotide primers in separate controls. The transfection efficiency was about 78.2 ± 6.3% (mean ± SD), which was considered to be adequate for the functional analyses. The human miR-1 miRNA was also used as a positive control. In this control, the human miR-1 miRNA mimic effectively down-regulated the expression of twinfilin-1 (also known as PTK9) by 80% at the mRNA level as detected by real-time PCR using TaqMan® Gene Expression Assays (Applied Biosystems) for PTK9. This positive control proved the effective delivery and activity of Pre-miR miRNA Precursor. We therefore used this model in further functional experiments. Each experiment was performed in triplicate.

## Competing interests

The authors declare that they have no competing interests.

## Authors’ contributions

WYL was responsible for experimental design, data analysis and drafting of the manuscript. ACMY performed the RNA extraction, miRNA expression profiling and real-time RT-PCR and ELISAs. KLKN performed the virus and cell cultures and virus infection experiments. LMS participated in editing the manuscript. SKWT, KFT and PKSC were responsible for design and supervision of the study. All authors read and approved the final manuscript.
